# Rare Double Primary Malignancies: A Pancreatic Gastrointestinal Stromal Tumor Mimicking as a Metastatic Lesion of Myoepithelial Carcinoma of Parotid Gland

**DOI:** 10.1155/2023/8274226

**Published:** 2023-12-26

**Authors:** Vania Myralda Giamour Marbun, Indah Jamtani, Ening Krisnuhoni, Sonar Soni Panigoro

**Affiliations:** ^1^Faculty of Medicine, Universitas Indonesia, General Surgery Department, Cipto Mangunkusumo Hospital, Diponegoro Street #71, Senen, Central Jakarta, Indonesia; ^2^Consultant of Oncology Division of General Surgery Department Faculty of Medicine, Universitas Indonesia, General Surgery Department, Cipto Mangunkusumo Hospital, Diponegoro Street #71, Senen, Central Jakarta, Indonesia; ^3^Staff of Pathology Anatomy Department, Faculty of Medicine, Universitas Indonesia, Pathology Anatomy Department, Cipto Mangunkusumo Hospital, Diponegoro Street #71, Senen, Central Jakarta, Indonesia; ^4^Staff of Digestive Division of General Surgery Department, Faculty of Medicine, Universitas Indonesia, General Surgery Department, Cipto Mangunkusumo Hospital, Diponegoro Street #71, Senen, Central Jakarta, Indonesia

## Abstract

Pancreatic gastrointestinal stromal tumors (PGISTs) are exceptionally rare, accounting for <5% of extra-gastrointestinal stromal tumors (EGISTs) and <1% of malignant pancreatic neoplasms. We present a unique case of concurrent double primary malignancies in a 46-year-old female with a history of recurrent myoepithelial carcinoma of the parotid gland, managed through surgical resection and adjuvant therapy. She presented with an enlarging abdominal mass, initially suggestive of pancreatic metastasis. Immunohistochemical analysis revealed positive staining for smooth-muscle actin (**SMA**) and **CD34** in both parotid and pancreatic tissues. Importantly, **CD117** expression was confined to the pancreatic tissue, confirming the diagnosis of PGIST rather than metastasis. Subsequently, a splenic-sparing distal pancreatectomy was performed, followed by immediate imatinib therapy. This case underscores the potential for the coexistence of rare primary malignancies with unique histopathological characteristics and organ involvement. When encountering a newly developed lesion in a distant organ, surgeons must consider the possibility of metastasis to guide therapeutic decision-making. Early diagnosis and appropriate intervention are paramount, particularly in the case of PGIST, given its infrequent presentation and clinical complexities.

## 1. Introduction

Myoepithelial carcinoma of the parotid gland (MCPG) is a biphasic tumor exhibiting a low potential of malignancy and a 25% chance of distant metastasis [1]. Until recently, extant literature has not explicitly detailed the pancreas as a recognized site of distant metastasis in MCPG cases. Conversely, the occurrence of pancreatic gastrointestinal stromal tumor (PGIST) is an exceedingly rare phenomenon, accounting for less than 5% of extra-gastrointestinal stromal tumors (EGIST) and less than 1% of the collective malignant pancreatic tumors [2, 3]. In the context of the synchronous or metachronous co-occurrence of a pancreatic lesion alongside a primary tumor originating from a different organ, the initial consideration should be given to the possibility of metastasis to the pancreas. It is noteworthy that this occurrence is relatively rare, comprising a mere 2% of all cases of organ metastases. Subsequently, an evaluation for other primary tumor types, including neuroendocrine tumors and gastrointestinal stromal tumor (GIST), should be undertaken [4]. In this research contribution, previously published as an abstract, we present a clinical case exemplifying the simultaneous emergence of two primary malignancies, MCPG and PGIST, underpinned by physical examination, comprehensive radiological documentation, and meticulous pathological specimen analysis [5].

## 2. Case Presentation

A 46-year-old female was referred to our digestive surgery service due to the presence of an abdominal mass that had been progressively growing over the course of the past two years. Her medical history revealed a recurrent MCPG spanning six years, which had undergone multiple surgical resections, including an extended radical parotidectomy performed by a surgical oncologist four years prior. Notably, the patient's medical history indicates an absence of familial cancer predisposition. In addition, she had received multiple rounds of chemotherapy comprising doxorubicin-paclitaxel-carboplatin-leucovorin-5-fluorouracil, along with two cycles of twenty-five rounds and thirty-three rounds of radiotherapy, resulting in a partial therapeutic response.

**Figure 1:**
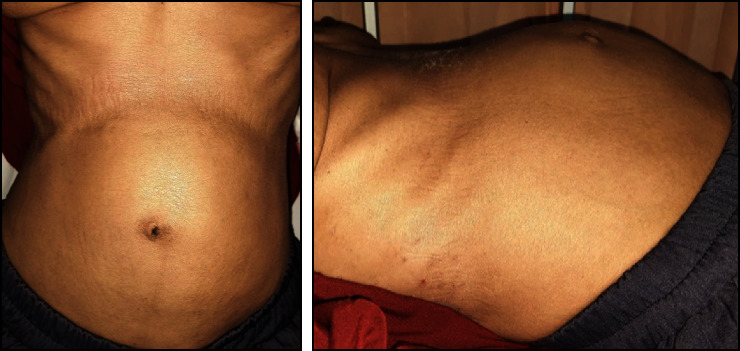
Prominent egg-shaped mass found on abdominal examination.

**Figure 2:**
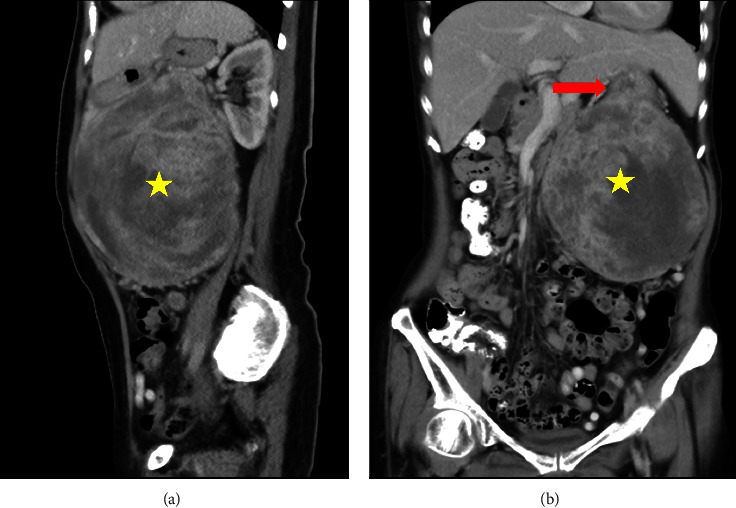
(a) Sagittal view. (b) Coronal view. Left-upper-quadrant heterogenous mass with central necrotic (yellow star); stomach with ill-defined margin to the mass (red arrow).

**Figure 3:**
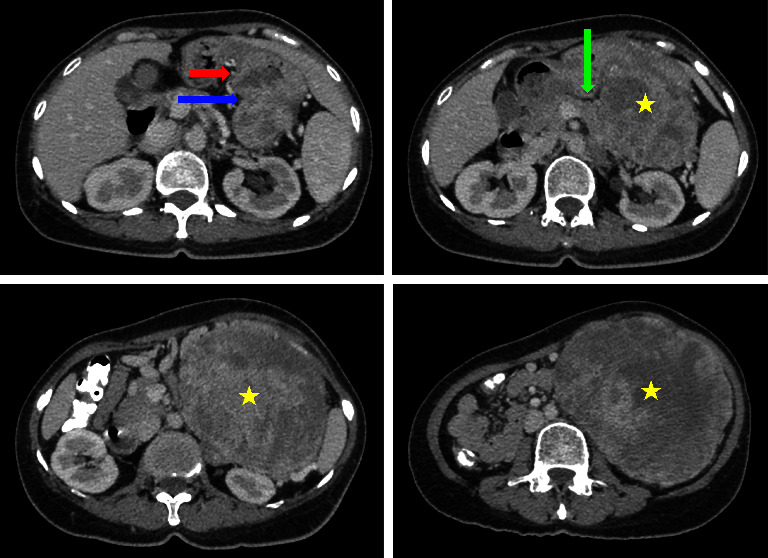
Axial view. Stomach (red arrow); ill-defined border of stomach to the mass (blue arrow); body of the pancreas (green arrow); left-upper-quadrant heterogenous mass with central necrotic (yellow star).

During the course of her treatment, the patient experienced a significant weight loss of twelve kilograms and noted the development of an egg-shaped mass in the upper left abdomen. Despite the absence of specific symptoms, she initially chose not to address the abdominal mass while continuing her therapeutic regimen for parotid gland cancer. Subsequently, the abdominal mass displayed progressive growth and became more conspicuous in tandem with her weight loss, as illustrated in Figure 1. Given this clinical presentation, an abdominal computed tomography (CT) scan with contrast was promptly ordered, revealing the presence of a heterogenous intraabdominal mass measuring 12.7 × 13.2 × 14 cm in size, situated in the left upper quadrant of the abdomen and presumptively originating from the posterior aspect of the stomach, as depicted in Figures 2 and 3. This mass exhibited necrotic components and extended anteriorly to involve the parietal peritoneum, exerting compressive effects on adjacent structures, including the pancreas, abdominal aorta, and vena cava, in a medial direction.

Consultations with radiologists yielded insights indicating the likely origin of the mass, which was presumed to arise from either the posterior surface of the stomach or the mesentery closely associated with the body of the pancreas. The radiological features of the mass suggested a mesenchymal or sarcoma-like tumor. The CT scan further revealed the close proximity of the tumor to adjacent structures, including the pancreas, although it did not exhibit overt infiltration into major vascular structures. Typically, a mesenchymal tumor originating from the stomach would raise suspicion of a gastrointestinal stromal tumor (GIST) of gastric origin. However, given the patient's complex medical history, a differential diagnosis was considered, encompassing both metastatic cancer to the pancreas and the primary cancer of gastric GIST. Subsequently, a surgical strategy was meticulously devised and implemented, aiming to excise the gastric mass while also acknowledging the potential necessity for a distal pancreatectomy.

**Figure 4:**
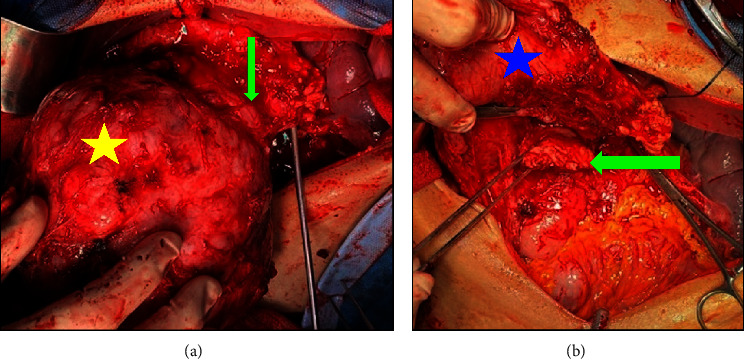
Intraoperative findings. (a) Mass (yellow star); pancreas (green arrow). (b) Stomach (blue star); pancreatic remnant (green arrow).

**Figure 5:**
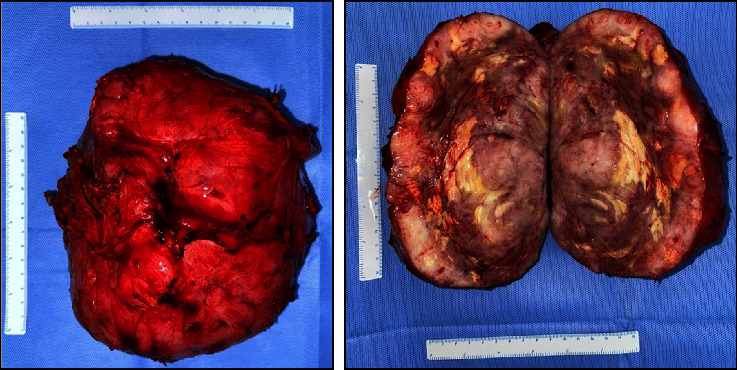
Tumor specimen.

**Figure 6:**
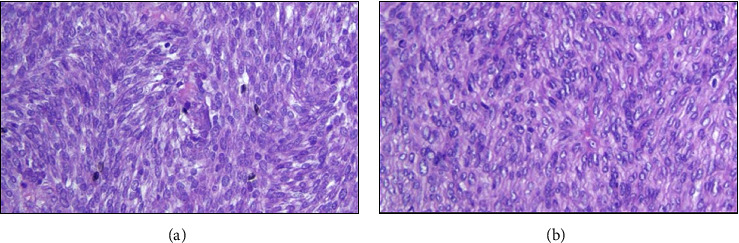
(a) Parotid (**H&E**). (b) Pancreas (**H&E**).

**Figure 7:**
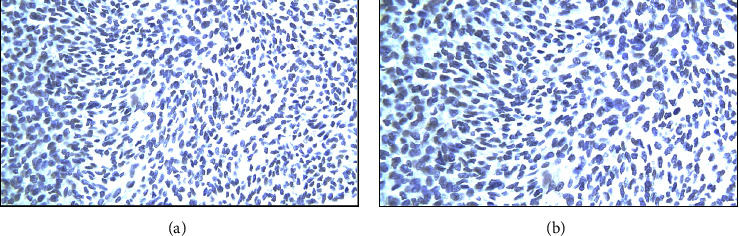
(a) Parotid (negative **DOG1**). (b) Pancreas (negative **DOG1**).

**Figure 8:**
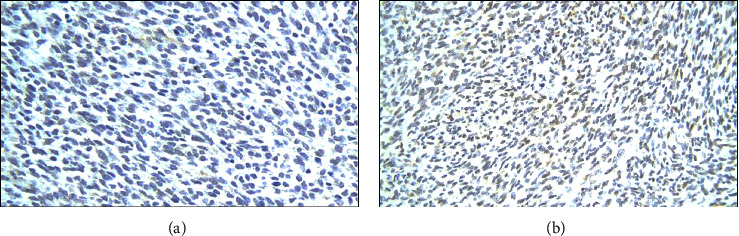
(a) Parotid (negative **CD117**); (b) pancreas (positive **CD117**).

**Figure 9:**
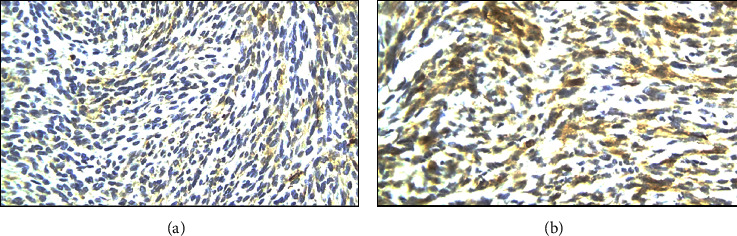
(a) Parotid (positive **SMA**); (b) pancreas (positive **SMA**).

**Figure 10:**
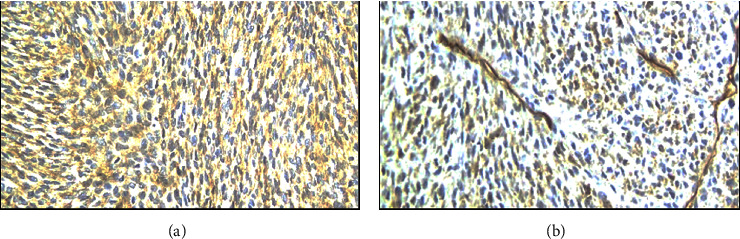
(a) Parotid (positive **CD34**); (b) pancreas (positive **CD34**).

Intraoperatively, the precise anatomical origin of the mass was unequivocally determined, localizing it to the pancreatic body and tail. Minimal adhesions were noted, and notably, there was no evidence of invasive infiltration in adjacent structures, including the stomach and major blood vessels, as depicted in Figure 4. Subsequent therapeutic measures involved the meticulous removal of the mass through a splenic-sparing distal pancreatectomy, executed with proficiency and without encountering complications. The gross morphological appearance of the tumor strikingly resembled that of a gastrointestinal stromal tumor (GIST), as visually exemplified in Figure 5. This resemblance was further substantiated by the conclusive pathology report, confirming the diagnosis of pancreatic GIST through comprehensive evaluation, as presented in Figures 6–10 and Table 1. Figures 6(a) and 6(b) provide a visual representation of the contrasting morphological characteristics exhibited by myoepithelial cells within the parotid gland in comparison to mesenchymal (spindle-shaped) cells present in the pancreas.  Figure 8(b) highlights the presence of positive **CD117** staining, an immunohistochemical marker for GIST, in the pancreas.

The postoperative period was characterized by its uneventful nature. She demonstrated a favorable recovery trajectory, ultimately resulting in discharge on the sixth day following the surgical procedure, devoid of any observed complications during the hospitalization. Upon subsequent follow-up in the outpatient clinic, the patient's therapeutic regimen included the initiation of imatinib at a daily dosage of 400 mg, approximately one-month postsurgery. Six months following the surgical intervention, the patient experienced recurrent tumor growth in the jaw region mandating the initiation of multiple rounds of cisplatin-based chemotherapy. Unfortunately, the concurrent administration of oral imatinib alongside intravenous cisplatin-based chemotherapy was halted due to onset of debilitating side effects, notably fatigue and breathlessness. Patient's health deteriorated, ultimately leading to her demise from pneumonia approximately four months afterward.

## 3. Clinical Discussion

This report, following Surgical Case Report (SCARE) guidelines, presents a unique clinical scenario involving the co-occurrence of a pancreatic gastrointestinal stromal tumor (PGIST) in a patient previously diagnosed with recurrent myoepithelial carcinoma of the parotid gland (MCPG) during the course of therapy [6]. The rarity of PGIST, particularly when it synchronously presents with a primary tumor as rare as MCPG, underscores the potential diagnostic challenges surgeons may encounter. In such cases, the identification of an asymptomatic, singular, and large mass exhibiting distinct radiological features becomes pivotal. Such features may include a well-circumscribed lesion displaying central necrosis and calcification, as discerned through an abdominal CT scan [3, 7–9]. These radiographic findings may raise suspicion of either pancreatic metastasis or pancreatic GIST. Consequently, when a patient with a history of cancer presents with such a pancreatic lesion, the initial consideration often gravitates toward pancreatic metastasis, followed by alternative etiologies, such as pancreatic neuroendocrine tumors of PGIST.

The appropriateness of surgical intervention in the context of secondary pancreatic cancer warrants thoughtful deliberation. Notably, curative resection remains a viable option for patients harboring a solitary metastatic lesion and possessing a favorable performance status [4]. Indeed, long-term survival outcomes have been reported following pancreatic resection for metastatic disease in MCPG [10].

Conversely, the surgical role in PGIST diagnosis and management is unequivocal. Our center opts against performing biopsies when GIST is suspected, primarily to mitigate the risk of tumor rupture and needle tract seeding, particularly when resection remains imperative. Pathognomonic radiological findings, such as heterogenous enhancement, exophytic growth, and the presence of a necrotic center, serve as valuable diagnostic clues for GIST without necessitating biopsy [11].

Immunohistochemical staining for **CD117**, also known as KIT/receptor *tyrosine kinase*, is a crucial diagnostic tool. **CD117** is expressed by the interstitial cell of Cajal (ICC), the progenitor cell of GIST, and serves as a highly sensitive and specific marker for GIST [12, 13]. However, it is essential to note that while **CD117** may also exhibit immunohistochemical expression in MCPG, it cannot be relied upon as definitive marker for diagnosing specific salivary gland tumors [14, 15]. The presence of positive **CD117** staining (Figure 8(b)) exclusively within the pancreatic tissue unequivocally confirms the diagnosis of PGIST, thereby distinguishing it from a metastatic lesion. Alternatively, the confirmation of MCPG diagnosis can be achieved through the identification of positive staining for smooth-muscle actin (**SMA**) and **p63**, as visually depicted in Figure 9 and detailed in Table 1. These specific immunohistochemical markers serve as reliable indicators for the presence of MCPG [16, 17].

**CD34** can be considered a dual-role marker, as it exhibits expression in both PGIST and MCPG. It is commonly used as a marker to assess microvessel density (MVD) in various tissues and tumors. In the context of PGIST, its expression is observed in more than 50% of cases [18]. It serves as one of the immunohistochemical markers used to aid in the diagnosis of GIST, often in conjunction with other markers like **CD117** and **DOG1**. On the other hand, **CD34** is also detected in more than 40% of MCPG [19, 20]. While not a specific marker for myoepithelial carcinoma, it can contribute to the characterization of these tumors when evaluated alongside other relevant immunohistochemical markers like **SMA** and **p63**.

This case emphasizes the intricate diagnosis considerations and pivotal role of radiological and immunohistochemical markers in discerning between PGIST and metastatic lesions, ultimately guiding appropriate therapeutic strategies.

## 4. Conclusion

This case serves as an exemplar of the potential occurrence of dual primary malignancies, characterized by the convergence of rare histopathological profiles and the involvement of distinct anatomical organs. When confronted with the presence of a newly developed lesion in a remote organ, surgeons face a critical diagnostic task: to meticulously rule out metastatic disease. It is important to emphasize that this diagnostic pursuit is not undertaken with the intent of bypassing surgical intervention but rather to provide the basis for informed decisions regarding the necessity of tailored adjuvant therapeutic strategies.

The intricate nature of managing dual primary malignancies underscores the importance of precision medicine and personalized treatment approaches. Collaboration among healthcare professionals from diverse specialties is essential in navigating the complex terrain of rare histopathological combinations and multiorgan involvement. This case highlights the evolving landscape of oncological care, emphasizing the need for tailored approaches to optimize patient outcomes in the context of concurrent malignancies.

## Figures and Tables

**Table 1:** Summary of histopathology and IHC.

	Parotid	Pancreas
CD117	Negative	Positive
DOG1	Negative	Negative
EMA	Negative	Negative
P63	Positive	Negative
SMA	Positive	Positive
CD34	Positive	Positive
S100	Positive	Negative
NSE	Positive	—
Vimentin	Positive	—
Ki-67	20%	—
Conclusion	Myoepithelial Carcinoma	Gastrointestinal Stromal Tumor (mitosis >20/10 hpf)
