# Correction to “Rare Double Primary Malignancies: A Pancreatic Gastrointestinal Stromal Tumor Mimicking as a Metastatic Lesion of Myoepithelial Carcinoma of Parotid Gland”

**DOI:** 10.1155/carm/9785303

**Published:** 2026-07-29

V. M. G. Marbun, I. Jamtani, E. Krisnuhoni, and S. S. Panigoro, “Rare Double Primary Malignancies: A Pancreatic Gastrointestinal Stromal Tumor Mimicking as a Metastatic Lesion of Myoepithelial Carcinoma of Parotid Gland,” *Case Reports in Medicine*, no. 2023 (2023), https://doi.org/10.1155/2023/8274226.

In the article titled “Rare Double Primary Malignancies: A Pancreatic Gastrointestinal Stromal Tumor Mimicking as a Metastatic Lesion of Myoepithelial Carcinoma of Parotid Gland,” there were multiple errors. These errors are shown and corrected below:

Error in Figure **7**:

The image of the pancreatic tissue in panel (b) shared overlapping features with the parotid tissue shown in panel (a). This error was introduced by the authors during manuscript preparation and the corrected figure is shown below:

**Figure 7:**
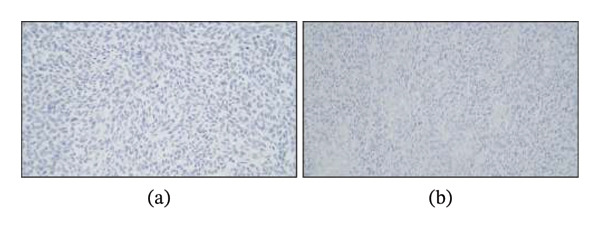
(a) Parotid (negative DOG1). (b) Pancreas (negative DOG1).

**Error in author affiliations:**

The affiliations of the listed authors V. M. G. Marbun, I. Jamtani, and S. S. Panigoro were incorrect. The corrected affiliations appear below:

V. M. G. Marbun^1^, I. Jamtani^2^, E. Krisnuhoni^3^, and S. S. Panigoro^4^

^1^Staff, Division of Digestive Surgery, General Surgery Department, Faculty of Medicine, Universitas Indonesia, General Surgery Department, Cipto Mangunkusumo Hospital, Diponegoro Street #71, Senen, Central Jakarta, Indonesia

^2^Faculty of Medicine, Universitas Indonesia, General Surgery Department, Cipto Mangunkusumo Hospital, Diponegoro Street #71, Senen, Central Jakarta, Indonesia

^3^Staff of Pathology Anatomy Department, Faculty of Medicine, Universitas Indonesia, Pathology Anatomy Department, Cipto Mangunkusumo Hospital, Diponegoro Street #71, Senen, Central Jakarta, Indonesia

^4^Consultant, Division of Surgical Oncology, General Surgery Department, Faculty of Medicine, Universitas Indonesia, General Surgery Department, Cipto Mangunkusumo Hospital, Diponegoro Street #71, Senen, Central Jakarta, Indonesia

We apologize for these errors.

